# How peer status and ability track shape behavioral disengagement over the transition from primary to secondary school

**DOI:** 10.1111/jora.13006

**Published:** 2024-08-07

**Authors:** Sofie J. Lorijn, Lydia Laninga‐Wijnen, Allison M. Ryan

**Affiliations:** ^1^ Department of Sociology University of Groningen Groningen The Netherlands; ^2^ Department of Developmental Psychology, INVEST Flagship University of Turku Turku Finland; ^3^ Combined Program in Education and Psychology University of Michigan Ann Arbor Michigan USA

**Keywords:** behavioral disengagement, peer status, school transition

## Abstract

The transition from primary to secondary school is often associated with an increase in behavioral disengagement, which undermines students' academic development. Prior studies examined the average development of behavioral disengagement across school transitions. This study examined how students' peer status in primary school and ability track in secondary school relate to trajectories of behavioral disengagement. We followed *n* = 1564 students who transitioned to secondary school across three time points: February/March, and May/June in students' final year of primary school and January/February, roughly 6 months after students transited to secondary school. Latent Growth Curve Analyses showed that on average, behavioral disengagement increased, but this increase mostly occurred *before* transitioning to secondary school. Peer status and track related to students' initial levels of behavioral disengagement, but not to their development in behavioral disengagement over the transition. Specifically, students who were viewed as more popular by peers, and students who ended up in the lowest track showed more behavioral disengagement in primary school, whereas students who were more accepted by peers were less disengaged in primary school.

## INTRODUCTION

Disengaged school behaviors, such as not paying attention in class and getting in trouble at school, undermine students' academic development. Students who regularly show disengaged school behavior face more difficulties later in life, including academic failure, school dropout, substance use, and delinquency (Bae, 2020; Henry et al., 2012). Despite the importance of being engaged in school, behavioral disengagement tends to increase in adolescence (Engels et al., 2017). The transition from primary to secondary school is generally thought to be the start of increasing behavioral disengagement. This transition coincides with the onset of adolescence: a developmental phase that is characterized by increased independence from adults, disrupting behaviors, and the need for peer status (Veenstra & Laninga‐Wijnen, 2023). Moreover, secondary schools may be less supportive to fit adolescents' developmental needs than primary schools, potentially explaining the increase in behavioral disengagement over the transition from primary to secondary school (Eccles et al., 1997). In line with this reasoning, some previous work detected an average increase of behavioral disengagement across school transitions (Benner & Wang, 2014; Eccles et al., 1997).

However, it is likely that students differ from each other with regard to the development of disengagement: not all students increase their behavioral disengagement (Fredricks et al., 2019). Two factors that may play a role in students' development of behavioral disengagement over the transition to secondary school are peer status and ability track. First, students' peer status in primary school may set the stage for the development of behavioral disengagement over the transition to secondary school. Previous work has shown the protective effect of being liked by peers on students' engagement (Ryan et al., 2019), and the negative impact of being rejected or popular (Engels et al., 2017). This study builds upon previous work by examining how students' peer status in primary school (i.e., being nominated by peers as popular, accepted, and rejected), lays a foundation for students' development of behavioral disengagement over the transition.

Second, students' ability track in secondary school may relate to their development in behavioral disengagement. In highly stratified secondary school systems, students are separated into different “tracks” for all subjects based on their ability (i.e., “explicit tracking” or “between‐school tracking”; OECD, 2016). Tracking is thought to greatly impact students' motivation and behavioral disengagement. For instance, anti‐school norms, lack of cognitive challenge, and increased sensitivity to ability comparisons may put students in lower academic tracks at risk for increased disengagement (Eccles et al., 1997; Trautwein et al., 2006). This study expands upon previous work by examining how ability tracks in secondary school relate to students' development of behavioral disengagement over the transition, in the highly stratified educational system of the Netherlands. Moreover, we study how peer status and ability tracking interact by examining if an increase in behavioral disengagement for popular students is more pronounced for students in the pre‐vocational track. Taken together, this study adds to the literature by examining how students' peer status in primary school and ability track in secondary school relate to trajectories of behavioral disengagement.

### The development of behavioral disengagement over school transitions

Behavioral disengagement reflects students' lack of engagement and school misbehavior. Behavioral disengagement may to some extent oppose behavioral engagement. For instance, not paying attention in class opposes listening carefully in class. Therefore, literature on behavioral engagement may inform us about behavioral disengagement. However, behavioral disengagement is more than the mere absence of engagement as it includes the presence of school misbehavior (Fredricks et al., 2019). Specifically, students who are engaged generally show low disengagement, but not being engaged does not necessarily imply disengagement. Therefore, although we mostly relied on engagement literature in our theoretical framework, we will study disengagement so as to include school misbehavior.

The transition from primary to secondary school is generally assumed to mark the onset of an increase in disengagement, for two main reasons. First, the transition from primary to secondary school coincides with the onset of adolescence. In adolescence, problematic behaviors including school disengagement and standing up against adults become more common. Displaying such behaviors can be a way for adolescents to show their maturity, as a means to close the gap between their social and biological maturation (Moffitt, 2018). Showing problematic behaviors in the presence of peers may be particularly attractive, as this may be rewarded with social status in adolescence (Veenstra & Laninga‐Wijnen, 2023). Thus, it is likely that behavioral disengagement in school increases at the onset of adolescence.

Second, the stage‐environment fit theory argues that the increase in behavioral disengagement in secondary school is driven by the worsened fit between students' developmental needs and the opportunities provided in the secondary school environment to fulfill these needs (Eccles et al., 1997). Specifically, early adolescents have an increased need for autonomy and self‐determination, the need for extending social relationships outside of their household, and are increasingly self‐conscious and concerned with ability comparisons (Eccles et al., 1997). Yet, secondary schools may be less well equipped to fit these needs compared with primary schools. Secondary schools are often more bureaucratic and teachers are confronted with larger and larger groups of students, resulting in school tasks being less tailored to individuals, and offering fewer opportunities for individual input (Midgley et al., 1989; Midgley & Feldlaufer, 1987). Moreover, the disruption in students' primary school peer network, and being confronted with multiple teachers in secondary school may complicate establishing high‐quality relationships with peers and teachers. Also, secondary school teachers are generally less friendly and caring compared with primary school teachers (Midgley et al., 1989). Furthermore, secondary schools are more focused on performance goals and ability comparisons, particularly in highly stratified systems. This mismatch between students' needs and the opportunities offered by their secondary school may underlie the increases in students' maladaptation.

More recent studies show a more nuanced view of youth' assumed social and academic maladaptation in lower secondary school (Brass et al., 2019). Students in secondary school showed better social adjustment compared with students in primary school, which aligns with the efforts of middle school reforms to promote social adjustment in middle schools in the USA (Brass et al., 2019). Yet, students in primary school were better academically adjusted compared with students in secondary school, for instance, showing higher behavioral engagement. Another recent study found that students' peer relationships improved after transitioning to secondary education, reasoning that the secondary school environment provides a better stage‐environment fit in the sense that a new peer group provides the opportunity to enhance peer status (Lorijn et al., 2024). Possibly, the secondary school environment may match students' needs for peer relationships while lacking to provide opportunities to fulfill students' academic needs. Therefore, whereas students' may socially adapt well to the secondary school environment, students' academic adaptation may lag behind. Taken together, the onset of adolescence and the gap between adolescents' academic needs and the opportunities offered in secondary school may increase students' behavioral disengagement. Therefore, *we expected a general increase in behavioral disengagement over the transition from primary to secondary school* (*Hypothesis 1*).

### Differences in disengagement trajectories

Beyond a possible average increase in behavioral disengagement over the transition from primary to secondary school, students greatly vary in their disengagement trajectories (Fredricks et al., 2019). For instance, in a previous study in the United States, the majority of students were stable in their moderate engagement from Grades 5 to 8 (62.2%), 18.4% remained highly engaged, 15.1% were temporarily disengaged, and 4.4% experienced problematically steep increases in disengagement (Li & Lerner, 2011). Likewise, students varied in their development of attendance (being present at school) from middle to high school, with most students (44%) maintaining their attendance trajectory despite the transition (Benner & Wang, 2014). Moreover, students' behavioral engagement in primary school relates to behavioral engagement in secondary school (*r* = .55; Engels, Colpin, et al., 2019; Engels, Pakarinen, et al., 2019), suggesting that students remain relatively stable in their level of behavioral disengagement compared to their peers. Taken together, this shows that school transitions may not increase disengagement for all students, expanding upon the prevailing belief of a general increase in disengagement. Few studies examined the different characteristics associated with different engagement trajectories. For instance, girls, white students, and students from higher income families were overrepresented in the high and moderate stable engagement groups (Li & Lerner, 2011). Taken together, differences in development in behavioral disengagement may be expected. Beyond students' background characteristics, students' peer status in primary school and students' ability track in secondary school may partially explain differences in disengagement trajectories over the transition from primary to secondary school.

### The role of prior peer status in disengagement trajectories

Peers are an important part of adolescents' social context at school, greatly impacting students' behavioral disengagement (Ryan et al., 2019). Peer relationships in school may include acceptance, rejection, and popularity. Acceptance reflects to what extent the student is liked by classmates, while rejection reflects to what extent the student is disliked by classmates (Veenstra et al., 2018). Popularity refers to students' prestige and reflects to what extent the student is regarded as “popular” or “cool” by classmates (Cillessen & Marks, 2011). As such, popularity is distinct from acceptance because it assumes a hierarchy by which students can be ranked (Cillessen & Marks, 2011). Moreover, whereas acceptance is associated with positive behaviors such as prosociality, popularity can also be associated with negative behaviors such as aggression. Thus, popular students are not necessarily liked by classmates. Students who are accepted by classmates may disengage less, while students who are rejected or popular in class may disengage more (Ryan et al., 2019).

Well‐liked (i.e., accepted) students tend to be more motivated in school, like school better, receive more academic support, and are more comfortable in the classroom, which benefits their academic achievement (Kiuru et al., 2020; Wentzel, 2005; Wentzel et al., 2021). The impact of being liked on students' engagement, and particularly disengagement, is less well documented. Students who are more accepted by peers are more engaged in primary school (De Laet et al., 2015), and are viewed by peers as more academically oriented (i.e., work hard and get good grades) across the transition from primary to secondary school (Brass & Ryan, 2021). Moreover, students who were accepted by peers participated more in extracurricular activities (i.e., behavioral engagement) over secondary school (Wang & Eccles, 2012). Yet, well‐liked students were more engaged at the start of secondary school, but students' acceptance did not matter for their behavioral *dis*engagement at the start of secondary school, as well as in the rest of secondary school (Engels et al., 2017). Thus, although likeability may be more strongly associated with engagement than with disengagement (Engels et al., 2017), the literature points to a possible protective effect of being liked for students' disengagement. Moreover, likeability is relatively stable across this school transition, and is related to academic effort in primary‐ as well as secondary school (Brass & Ryan, 2021). Possibly, students who were liked in primary school may start secondary school with pro‐school attitudes and higher school well‐being, protecting them from increasing disengagement. Taken together, *we expected that students with higher levels of acceptance in primary school would have lower levels of behavioral disengagement at the end of primary school, and less steep increases in behavioral disengagement over the transition from primary to secondary school as compared to students who were less accepted* (*Hypothesis 2*).

Students who are rejected by peers show more undesirable classroom behavior, are less engaged, show less interest in schoolwork, and are more at risk for decreased classroom participation, absenteeism, and school dropout (Buhs & Ladd, 2001; DeRosier et al., 1994; Furrer & Skinner, 2003; Wentzel & Asher, 1995). Being rejected by peers in primary school may set the stage for increasing disengagement in secondary school. Peer rejection in primary school can have long‐term negative effects on students' educational outcomes (Lorijn et al., 2022). Students who were rejected in primary school may thus further increase in their disengagement into secondary school. Moreover, for students who were rejected in primary school, entering a new peer group facilitates an opportunity to enhance their peer status (Kinney, 1993), and disengaging in school may be a way to gain this status (LaFontana & Cillessen, 2010; Veenstra & Laninga‐Wijnen, 2023). Consequently, *we expected that students with higher levels of rejection in primary school would have higher levels of behavioral disengagement at the end of primary school, and steeper increases in behavioral disengagement over the transition from primary to secondary school as compared to students who were less rejected* (*Hypothesis 3*).

Popular students may be at risk for increased disengagement. In primary school, popularity may not yet be distinguished from likability (Lorijn et al., 2023), and popular students may often be well‐liked and less behaviorally disengaged. Yet, in secondary school, peer status may become more crystallized, and disengaged school behaviors may be respected as “cool” and provide status (Veenstra & Laninga‐Wijnen, 2023). Students who are popular in primary school often regain this popularity in secondary school (Brass & Ryan, 2021). Thus, increasing their behavioral disengagement may be a way for students who were popular in primary school to regain their status in secondary school. In line with this argumentation, students' strive for status peaks directly after the transition to secondary school, when students renegotiate their relative status (Dawes & Xie, 2017). Therefore, particularly at the start of secondary school, students may show disengaged behaviors that will help them to maintain their popular status (De Laet et al., 2015; Engels et al., 2017; LaFontana & Cillessen, 2010). Taken together, *we expected that students with higher levels of popularity in primary school would have lower levels of behavioral disengagement at the end of primary school, and steeper increases in behavioral disengagement over the transition from primary to secondary school as compared to students who were less popular* (*Hypothesis 4*).

### The role of ability grouping in disengagement trajectories

Ability grouping (i.e., tracking) is a strategy applied in many educational systems to provide students with schooling adjusted to their level of education, to correctly allocate them to future educational and vocational careers. Furthermore, ability tracking aims to create more homogeneous groups, permitting a focused curriculum and tailored instruction with less need for differentiation. Most countries have educational systems with some form of ability grouping (Bol & Van de Werfhorst, 2013). The Dutch school system is highly stratified, separating students into different “tracks” for all subjects when they enter secondary school when students are roughly 12 years old (OECD, 2016). Based on students' academic ability, they are tracked in pre‐vocational (VMBO), general (HAVO), and pre‐university education (VWO).

Students' ability track may relate to their development of behavioral disengagement. Particularly students in the lowest and highest track may experience impact of ability tracking on their behavioral disengagement, as compared to students in general tracks. Education can provide legitimation, conferring success to some and failure to others (Meyer, 1977). In this system, students in higher tracks are more privileged and face higher societal educational expectations, whereas students in lower tracks are stigmatized as valued less in society (Meyer, 1977; Spruyt et al., 2015; Van Houtte & Stevens, 2008). Students in general tracks are “in between” these levels and may be less likely to face high societal expectations or to be stigmatized. Moreover, the level of education offered to the general track may be most similar to the level of education in primary school. Thus, whereas students in the general track may experience less impact of ability tracking on their status and level of education, students in other tracks may experience a larger impact which in turn affects their behavioral disengagement.

For students in pre‐vocational tracks, different scenarios may be at play. In primary school, lower achieving students may disengage because they cannot keep up. On the one hand, tracking these students into the pre‐vocational track should partially bridge the discrepancy between students' performance and their level of education, decreasing their behavioral disengagement. Moreover, while adolescents' sensitivity to social comparison increases, students in lower tracks generally have a higher academic self‐concept (Belfi et al., 2012). This can be explained by the big‐fish‐little‐pond effect (BFLPE): equally able students have a higher academic self‐concept in lower tracks compared to if they would be in a higher track because students compare themselves with peers in their immediate environment (Marsh et al., 2008; Trautwein et al., 2006). Higher self‐concept may in turn lower students' behavioral disengagement (Skinner et al., 2008).

On the other hand, tracking students on their ability may impact their status and sense of success because not meeting the achievement criteria of the educational system can be considered as ‘failing’. In turn, students may reject the current system, resulting in anti‐school attitudes in lower tracks and pro‐school attitudes in higher tracks (Van Houtte, 2006). Therefore, ability tracking may relate to polarization in school attitudes (Hargreaves, 1967). Previous studies found that students in lower tracks are generally more disengaged, showing more school misconduct and less effort in school (Carbonaro, 2005; Van Houtte, 2017; Van Houtte & Stevens, 2008). Additionally, students in lower tracks are often instructed by less qualified teachers, offered less cognitively challenging instructional strategies, and confronted with lower teacher expectations (Andersen, 2018; Toledo Román & Valenzuela, 2015; Van Houtte, 2006). Taken together, *we expected that students in pre‐vocational tracks would have higher levels of behavioral disengagement at the end of primary school as compared to students in other tracks* (*Hypothesis 5*), *and explored how their behavioral disengagement would develop over the transition from primary to secondary school, as compared to students in the other tracks*.

Students in the pre‐university track may be less disengaged in primary as well as secondary school, compared to students in lower tracks. These students may achieve higher in primary school, increasing their motivation (Engels, Colpin, et al., 2019; Engels, Pakarinen, et al., 2019), and lowering their disengaged behaviors in primary school. When students' secondary school track is determined at the end of primary school, students who are perceived by teachers as showing school‐appropriate behaviors (i.e., being motivated to learn, following teachers directions, and sticking to class rules) are more likely to receive higher teacher track recommendations (Sneyers et al., 2018; Timmermans et al., 2016). When moving to the pre‐university track in secondary school, students may remain to have low disengagement because the pre‐university track is a good fit for their needs. Particularly in the highest track, students are more cognitively challenged, fitting adolescents' developmental needs (Eccles et al., 1997). Furthermore, the differentiation–polarization effect puts these students in a classroom with pro‐school attitudes and pro‐academic norms. These students may experience status from being on the highest track, for instance promoting their wellbeing (Belfi et al., 2012). Students in higher tracks may like school more and may find education more important. Taken together, *we expected that students in the pre‐university track would have lower levels of behavioral disengagement at the end of primary school, and would have less steep increases in disengagement over the transition from primary to secondary school as compared to students in other tracks* (*Hypothesis 6*).

### Popular students in pre‐vocational tracks

The expected increase in behavioral disengagement for popular students may be more pronounced for students in the pre‐vocational track. From secondary school onwards, popularity becomes more crystallized, and prosocial, well‐liked popular students, divide from aggressive popular students (van den Berg et al., 2020). Showing disengaged school behavior may be a more negative, aggressive way to gain popularity. Which kind of behavior (i.e., prosocial or aggressive) leads to status gain, depends on the school culture and the popularity norm in the classroom (Laninga‐Wijnen et al., 2017). Aggressive popularity norms may be more prevalent in vocational tracks, because these tracks more often develop anti‐school attitudes (Van Houtte, 2006), aggressive popularity norms may be more prevalent in these tracks. For instance, not only did students in lower educational tracks consume more alcohol and smoke more, alcohol and smoking more strongly related to popularity in lower educational tracks (Peeters et al., 2021). Moreover, absenteeism is related to popularity, particularly in cliques with anti‐school values (Cillessen & van den Berg, 2012). Thus, showing disengaged school behavior may be a way to gain popularity in pre‐vocational tracks in particular. Therefore, *we expected that the increase in behavioral disengagement over the transition from primary to secondary school for popular students would be steeper for students in the pre‐vocational track, as compared to popular students in the other tracks* (*Hypothesis 7*).

### The present study

This study examined how students' peer status in primary school and ability track in secondary school relate to students' behavioral disengagement at the end of primary school, and over the transition from primary to secondary school. Following the stage‐environment fit theory, we expected a general increase in behavioral disengagement over the transition. Beyond an average increase, we argue that students differ in their development of behavioral disengagement. Specifically, we expected that students' peer status in primary school and ability track in secondary school would relate to their behavioral disengagement. Regarding peer status, we expected that acceptance would have a protective effect, whereas rejection and popularity may pose students at risk for high behavioral disengagement. Focusing on ability track, we expected that students in lower track levels are more at‐risk for behavioral disengagement. We expected an interaction effect between peer status and ability track, such that the increase in behavioral disengagement over the transition for popular students would be steeper for students in the pre‐vocational track as compared to popular students in the other tracks. Table 1 provides a full overview of our hypotheses. Our hypotheses, methods, and analysis plan were preregistered at the Open Science Framework (OSF) (see https://osf.io/5tqw2).

**TABLE 1:** Overview of hypotheses.

Hypothesis		End of primary school (intercept)	Over the transition from primary to secondary school (slope)
1	Average		Increase in behavioral disengagement
2	Peer status	Students with higher levels of *acceptance* in primary school would have lower levels of behavioral disengagement than students who are less accepted	Students with higher levels of *acceptance* in primary school would have less steep increases in behavioral disengagement over the transition than students who are less accepted
3		Students with higher levels of *rejection* in primary school would have higher levels of behavioral disengagement than students who are less rejected	Students with higher levels of *rejection* in primary school would have steeper increases in behavioral disengagement over the transition than students who are less rejected
4		Students with higher levels of *popularity* in primary school would have lower levels of behavioral disengagement than students who are less popular	Students with higher levels of *popularity* in primary school would have steeper increases in behavioral disengagement over the transition than students who are less popular
5	Ability track	Students in *pre‐vocational* tracks would have higher levels of behavioral disengagement than students in other tracks	[Explored]
6		Students in the *pre‐university* track would have lower levels of behavioral disengagement than students in other tracks	Students in the *pre‐university* track would have less steep increases in disengagement over the transition than students in other tracks
7	Popular pre‐vocational students	[No hypothesis]	The increase in behavioral disengagement over the transition for popular students would be steeper for students in the pre‐vocational track than for popular students in the other tracks

## METHODS

### Procedure and participants

We used data from the PRIMS project (an acronym for the transition from PRIMary to Secondary education). PRIMS aimed to study the role of peers in adolescent adjustment across the transition from primary to secondary school, and follows participants over this transition. Data were collected in February–March 2021 (T1) and May–June 2021 (T2) in primary school, and in January–February 2022 (T3), roughly 6 months after students transitioned to secondary school (for more information, see Zwier, Geven, et al., 2023; Zwier, Lorijn, et al., 2023). The first survey was collected before, and the second survey after students took the standardized exit test and received their final teacher track recommendation for secondary school. The third survey was conducted after a longer interval to ensure students were settled into their peer groups, allowing for a more reliable measurement of their peer status. We selected schools using a stratified sample design, resulting in a representative sample on region, level of urbanization, socio‐economic composition, denomination, school size, and students' track recommendations at the school level. All 2662 students in their last year of primary school (Dutch Grade 8) of the selected schools were invited to participate, of whom 1693 (63.6%) received active, written parental consent to participate. In total, 1643 out of these 1693 students (97.1%) filled out the first survey. Given that 51 students joined the PRIMS project for the second survey, the total sample size was *N* = 1694 students. 1634 students participated in the second survey, of which 1583 (96.3%) also participated in the first survey. Students filled out the first and second surveys online during school hours under the supervision of their teacher, which took about 45 min. A total of 741 students who participated in the first and second survey, also participated in the third survey (T3), after they transitioned to secondary school. Students filled out the third online survey at home, which took about 15 min.

There were two types of missing data in this study. First, attrition reduced the number of participants from 1643 in the first survey to 741 in the third survey. Second, we selected only classrooms with a minimum of 40% participation rate at T1 to more accurately and reliably estimate students' peer status (Marks et al., 2013). This further decreased the sample from 741 to 698 students. Missing data due to attrition is assumed to be missing at random (MAR) and were handled using the full information maximum likelihood estimate (FIML), which is an unbiased method for handling missing data at random (Muthén & Muthén, 2017). Applying FIML, we used the total sample of *N* = 1694 students to select classrooms with a minimum participation rate of 40%, resulting in a sample size of 1564 students from 98 classes in 71 schools.

A robustness check on handling missing data by repeating the analysis using listwise deletion (*n* = 689) showed similar results compared with the results when using FIML (*n* = 1564). The sole difference is the average trajectory of behavioral disengagement, showing no change in behavioral disengagement over time when using listwise deletion, as indicated by the insignificant slope (*M*_*S*_ = 0.004 [−0.001; 0.008], *p* = .101). This may be explained by the subsample of 689 students being selective on behavioral disengagement by having lower behavioral disengagement.

### Measures

#### Behavioral disengagement (all time points)

We measured behavioral disengagement by the following six items of the Youth Self Report Checklist: “I goof off (act silly and put off schoolwork) during work time in class,” “I find reasons to get out of class,” “I do not follow school rules,” “I get in trouble at school,” “I zone out at school,” and “I do not pay attention in class” (Achenbach, 1991). Students rated the items as 0 = *never* to 3 = *always*. A scale was generated by averaging the answers on the items for every participant with a valid response to at least two items. The scale revealed an acceptable reliability score over the three time points (all α ≥ .72), which could not be improved by deleting an item. A confirmatory factor analysis (CFA) showed that for all time points, all variables loaded on the factors with *p* < .001. Model fit was good for the CFA correlating the same items over time (*χ* ^2^ (114, *N* = 1559) = 364.91, *p* < .001, RMSEA = 0.038, CFI = 0.959, TLI = 0.944, SRMR = 0.041). We assessed measurement invariance to check if the meaning of the behavioral disengagement scale was the same over time. We included configural, metric, and scalar steps, and included the correlations between the same items at different time points. The fit was acceptable to good for all steps indicating measurement invariance (Configural: *χ* ^2^ (114, *N* = 1559) = 364.91, *p* < .001, RMSEA = 0.038, CFI = 0.959, TLI = 0.944, SRMR = 0.041; Metric: *χ* ^2^ (124, *N* = 1559) = 385.75, *p* < .001, RMSEA = 0.037, CFI = 0.957, TLI = 0.947, SRMR = 0.045; Scalar: *χ* ^2^ (134, *N* = 1559) = 541.66, *p* < .001, RMSEA = 0.044, CFI = 0.933, TLI = 0.923, SRMR = 0.052). This means that the meaning of the behavioral disengagement scale was the same over time, and a trajectory can be estimated.

#### Peer acceptance, rejection and popularity (T1)

We assessed peer acceptance, rejection, and popularity using classroom‐based peer nominations. Using peer nominations is a commonly used and reliable way to assess peer status (Cillessen & Marks, 2017; Veenstra et al., 2018). We asked students: “Which classmates do you like?”, “Which classmates do you dislike?”, and “Which classmates are popular?” Students could nominate an unlimited number of classmates using the provided list showing all classmates' names. To account for differences in class size, the number of nominations received was divided by the number of possible nominations (i.e., the total number of classmates filling out the survey minus 1). This resulted in proportion scores ranging from 0 to 1, with more nominations indicating higher scores. We selected only classrooms with a minimum of 40% participation rate to give a more reliable view of students' peer status (Marks et al., 2013). We centered the peer status variables at the grand mean for all models.

#### Track level (T3)

We assessed students' track level in secondary school by asking: “What is your track level?” All track levels in the Netherlands, and combined tracks, were provided as answer categories (for more information, see Zwier, Geven, et al., 2023; Zwier, Lorijn, et al., 2023). The answer categories were recoded into three categories to test the confirmatory hypotheses 0 = *pre‐vocational track*; 1 = *general track*; 2 = *pre‐university track*. We chose to assign students in a combined track to the highest level of this track (e.g., students in a “pre‐vocational/general” track were coded as “1 = *general track*”) because students in combined tracks have access to resources of the higher track (e.g., higher quality teachers, pro‐school norms), and better opportunity to transfer to this higher track.

We recoded this variable into two dummy variables for our main analyses to test our hypotheses and to consider the ordinal nature of the scale. We created the dummy variables “General track” (0 = *pre‐vocational or pre‐university track*, 1 = *general track*) and “Pre‐university track” (0 = *pre‐vocational or general track*, 1 = *pre‐university track*), leaving the pre‐vocational track as the comparison group to best fit our hypotheses. In our main analyses, both dummy variables were included which means they are conditional on each other (i.e., contrast coding). For instance, the estimate for the “general track” compares students in the general track with students in the pre‐vocational track, as this is the estimate for students who are not in the pre‐university track (score a “0” on the other dummy variable “Pre‐university track”). We perform sensitivity analyses with different coding of the dummy variables. Although students are only tracked in secondary education, the LGCA generates an effect of students' track on their intercept of behavioral disengagement in primary school. Conceptually, we consider track to be an approximation for students' track advice in primary school. Thus, we interpret these results with caution by discussing different initial levels of disengagement for students who later end up in different tracks, without assuming a predictive effect of track.

#### Gender

We controlled for gender in the main analyses because boys generally behaviorally disengage more, particularly boys in lower tracks (Van Houtte, 2017). Students indicated whether they were a boy, a girl, or other. Because only two students (0.1%) indicated to be “other,” we coded “other” missing and gender as 0 = *boy*; 1 = *girl*. Boys thus are the reference group.

### Analytic strategy

**FIGURE 1:**
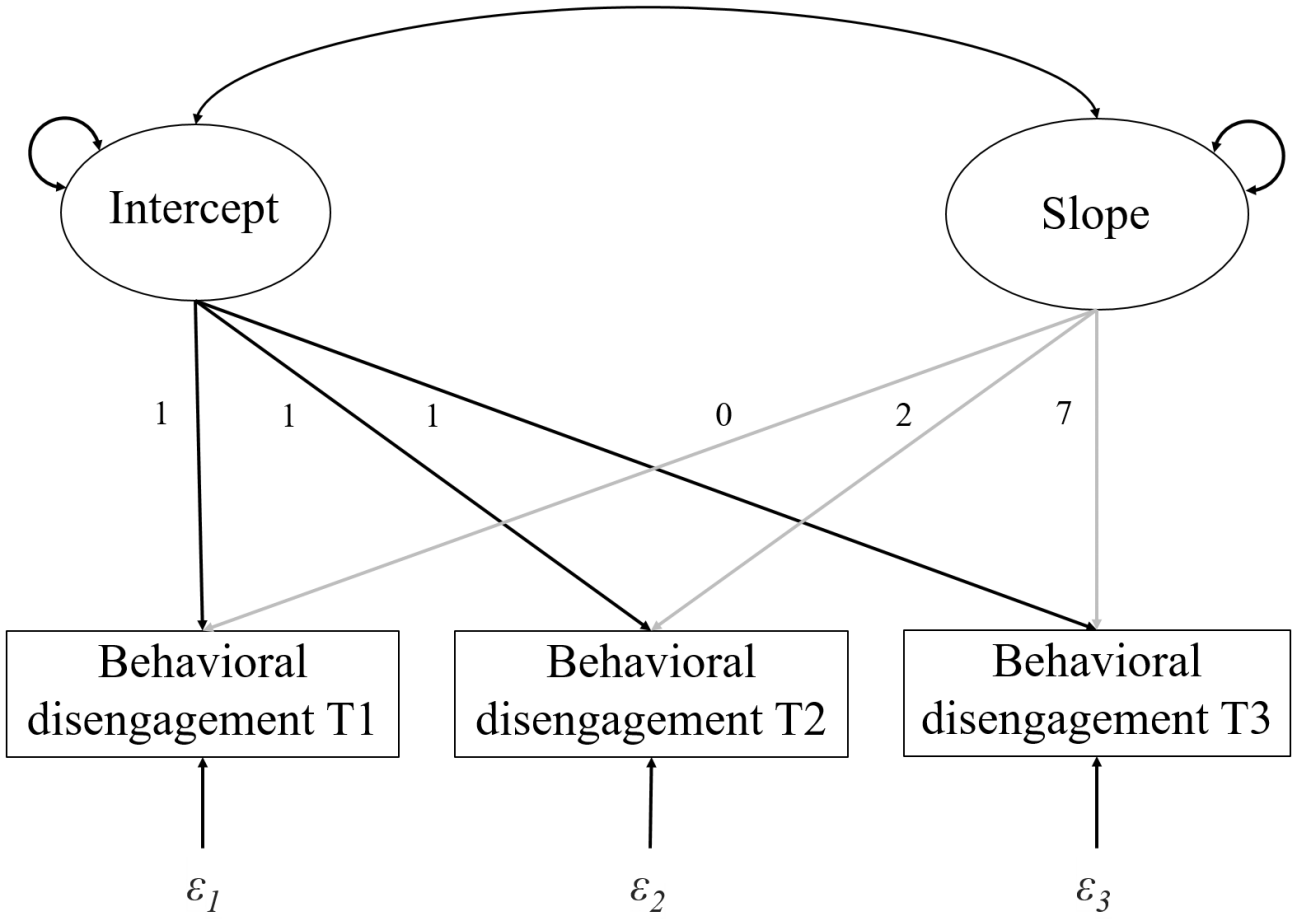
Latent growth curve model.

We performed two steps of analysis in M*plus* version 8.5 to test the hypotheses. First, we analyzed an unconditional model (i.e., without predictors) using Latent Growth Curve Analyses (LGCA). Figure 1 visualizes this latent growth curve model. This showed the general development of behavioral disengagement, testing hypothesis 1 that there would be a general increase in behavioral disengagement over the transition from primary to secondary school. Second, we analyzed a conditional LGCA model (i.e., with predictors of the intercept and slope) to examine the relation between the predictors and the development of behavioral disengagement, controlling for gender. With this analysis, we tested hypotheses 2–7, on the relations between peer status in primary school and track level in secondary school on students' development of behavioral disengagement over the transition from primary to secondary school. In all models, we controlled for clustering of students at the classroom level using the “complex analysis” option and “cluster” command in M*plus*. Model fit was assessed by evaluating the Root Means Square Error of Approximation (RMSEA), the Comparative Fit Index (CFI), the Tucker–Lewis Index (TLI), the standardized root mean squared residual (SRMR), and χ^2^ (Cheung & Rensvold, 2002). The RMSEA should not exceed 0.08 and shows a good fit for values of ≤0.06. For CFI and TLI, ≥0.90 is considered acceptable, and ≥ 0.95 reflects a good model fit. The SRMR of ≤0.06 is considered acceptable and ≤ 0.05 reflects a good fit. The loading of the slope factor for behavioral disengagement in Feb/March ‘21 (T1) was fixed to 0 so that the mean intercept reflects the level of behavioral disengagement at T1. For the measures in May/June ‘21 (T2), and Jan/Feb ‘22 (T3, secondary school), the loadings of the slope factors were fixed to 2 and 7, respectively, to reflect the unequal intervals between surveys. Appendix 1 describes testing of the assumptions for latent growth curve models.

## RESULTS

### Descriptive statistics

Table 2 presents the descriptive statistics for the behavioral disengagement and peer status variables by track level and gender. On average, behavioral disengagement was low and slightly increased from *M =* 0.45 halfway through students' last year of primary school, to *M =* 0.49 at the end of primary school, and slightly decreased to *M =* 0.46 in secondary school. Students in the pre‐vocational track had higher levels of behavioral disengagement in primary school, compared to students in the general track (T1: Wald test (2, 722) = 0.09, *p* = .036, *d* = 0.44; T2: Wald test (2, 722) = 0.06, *p* = .026, *d* = 0.45) and to students in the pre‐university track (T1: Wald test (2, 722) = 0.09, *p* = .036, *d* = 0.73; T2: Wald test (2, 722) = 0.06, *p* = .014, *d* = 0.77). Moreover, students in the pre‐vocational track varied more in their levels of behavioral disengagement in primary school, as indicated by the larger standard deviations. Behavioral disengagement was higher among boys than girls at T1 (Wald test (1506) = 0.048, *p* = .001; *d* = 0.22), and boys more often were rejected than girls (Wald test (1506) = 0.004, *p* = .046; *d* = 0.23).

**TABLE 2:** Descriptive statistics for the behavioral disengagement and peer status variables by track level and gender.

Variables	*N* ^a^ *M*	*M* (SD)	Group mean (SD)			Wald pre‐voc. vs. gen.	*p*	Wald pre‐voc. vs. pre‐uni.	*p*	Wald gen. vs. pre‐ uni.	*p*	Group mean (SD)		Wald	*p*
			Pre‐vocational (26.3%)	General (20.1%)	Pre‐university (53.6%)							Boys (50.1%)	Girls (49.9%)		
Behavioral disengagement T1	1492	0.45 (0.35)	0.50 (0.19)	0.43 (0.10)	0.40 (0.10)	0.09	.036	0.09	.036	−0.00	.806	0.49 (0.39)	0.41 (0.32)	0.05	.001
Behavioral disengagement T2	1489	0.49 (0.40)	0.54 (0.18)	0.47 (0.12)	0.43 (0.12)	0.06	.026	0.06	.014	0.00	.875	0.53 (0.40)	0.44 (0.37)	0.04	.074
Behavioral disengagement T3	689	0.46 (0.37)	0.52 (0.16)	0.47 (0.16)	0.42 (0.12)	0.01	.934	0.04	.293	0.04	.496	0.47 (0.39)	0.44 (0.35)	0.04	.303
Peer acceptance	1513	0.42 (0.19)	0.43 (0.04)	0.44 (0.04)	0.44 (0.04)	−0.00	.721	−0.00	.445	−0.00	.779	0.41 (0.19)	0.44 (0.20)	−0.00	.577
Peer rejection	1513	0.12 (0.13)	0.13 (0.02)	0.10 (0.01)	0.10 (0.02)	0.01	.073	0.00	.687	−0.00	.123	0.13 (0.14)	0.10 (0.12)	0.00	.046
Peer popularity	1513	0.10 (0.14)	0.11 (0.02)	0.11 (0.02)	0.10 (0.02)	0.00	.936	0.00	.966	−0.00	.910	0.11 (0.15)	0.10 (0.13)	0.01	.097

^a^ For the Wald‐tests on track, *n* = 722, for the Wald‐test on gender, *n* = 1506.

Table 3 presents the correlations of the behavioral disengagement and peer status variables. Students' behavioral disengagement remained relatively stable over time (all *r* ≥ .50). Being accepted by peers was weakly negatively related to behavioral disengagement (all *r* ≤ −.05), and rejection weakly positively related to behavioral disengagement (all *r* ≥ .10). Popularity weakly positively related to behavioral disengagement (all *r* ≥ .10). Acceptance and rejection were moderately negatively related (*r* = −.43). Acceptance and popularity were weakly positively related (*r* = .25), although acceptance weakly related to lower behavioral disengagement and popularity weakly related to higher disengagement.

**TABLE 3:** Correlations between behavioral disengagement and peer status (*N* = 1564).

	1.	2.	3.	4.	5.
**Behavioral disengagement**					
1. T1	—				
2. T2	0.68***	—			
3. T3	0.50***	0.59***	—		
**Peer status**					
4. Acceptance	−0.11***	−0.09***	−0.05	—	
5. Rejection	0.12***	0.12***	0.10**	−0.43***	—
6. Popularity	0.11***	0.12***	0.10**	0.25***	−0.02

** *p* ≤ .01.

*** *p* ≤ .001.

### Average development of behavioral disengagement

An unconditional latent growth model for behavioral disengagement was specified, revealing acceptable model fit *χ* ^2^ (1, 1558) = 17.08, *p* < .001, RMSEA = 0.102 [0.063; 0.146], CFI = 0.965, TLI = 0.896, SRMR = 0.026. The positive slope (*B*_*S*_ = 0.005 [0.000; 0.009], *p* = .034) indicated that students' average behavioral disengagement increased over time, in line with hypothesis 1. Appendix 1 displays a sensitivity analysis including a quadratic term in the model with the variance of this slope fixed as zero, showing similar results. Descriptive statistics and the model with the quadratic term revealed that behavioral disengagement mostly increased in primary school, and slightly recovered in secondary school. Significant variances around the intercept and slope (*D*_i_ = 0.11, *p* < .001; *D*_*s*_ = 0.002, *p* < .001) indicate that students vary in their initial levels and development of behavioral disengagement. Students with higher initial levels of behavioral disengagement had less rapid increases in behavioral disengagement over time, as indicated by the negative correlation between the intercept and slope (*r* = −.01, *p* < .001).

### Differences in disengagement trajectories

To examine the role of peer status and track level in students' behavioral disengagement development, we estimated three models to evaluate the relative addition of variables. The first model included gender and peer status variables (acceptance, rejection, popularity), the second model added track level, and the third model added the interaction between popularity and the pre‐vocational track. Model fit was good for all models, as shown in Table 4. Table 5 presents the parameter estimates for the three models. We interpreted the main effects in model 2, and the interaction effects in model 3.

**TABLE 4:** Model fit indices of the latent growth curve predictor models.

	*χ* ^2^	df	*p*	RMSEA	CFI	TLI	SRMR
Model 1	23.05	5	<.001	0.048	0.979	0.936	0.013
Model 2	24.98	7	.001	0.041	0.981	0.942	0.012
Model 3	29.54	9	.001	0.038	0.978	0.935	0.010

**TABLE 5:** Intercepts and slopes of the LGCA on behavioral disengagement by peer status and track level.

	Model 1		Model 2		Model 3	
	B intercept (SE)	B slope (SE)	B intercept (SE)	B slope (SE)	B intercept (SE)	B slope (SE)
Gender (0 = boy)	−0.059 (0.018)***	0.006 (0.004)	−0.063 (0.017)***	0.006 (0.004)	−0.063 (0.017)***	0.006 (0.004)
Acceptance	−0.194 (0.065)**	0.016 (0.009)	−0.200 (0.065)**	0.015 (0.009)	−0.203 (0.064)**	0.016 (0.009)*
Rejection	0.201 (0.100)*	0.009 (0.017)	0.166 (0.103)	0.008 (0.017)	0.157 (0.099)	0.008 (0.017)
Popularity	0.342 (0.075)***	−0.001 (0.013)	0.334 (0.073)***	−0.001 (0.013)	0.686 (0.232)**	0.015 (0.035)
General track			−0.059 (0.047)	0.001 (0.006)	−0.053 (0.047)	0.000 (0.006)
Pre‐university track			−0.097 (0.035)**	−0.001 (0.005)	−0.095 (0.036)**	−0.001 (0.005)
Popularity * General track					−0.757 (0.289)**	0.028 (0.049)
Popularity * Pre‐university track					−0.379 (0.304)	−0.040 (0.042)
*R* ^2^ Intercept	5.4%***		7.0%***		8.2%***	
*R* ^2^ Slope	0.8%		0.8%		1.6%	

* *p* ≤ .05.

** *p* ≤ .01.

*** *p* ≤ .001.

#### The role of prior peer status in disengagement trajectories

Students who were acceptance had slightly lower initial levels of behavioral disengagement at the end of primary school, whereas peer acceptance was unrelated to behavioral disengagement over the transition (*B*_*i*_ = −0.200 [−0.328; −0.072], *p* = .002, *β*_*i*_ = −.116; *B*_*s*_ = 0.015 [−0.001; 0.032], *p* = .071, *β*_*s*_ = .067), partly in line with hypothesis 2. The effect of acceptance on the intercept was small, with students who scored one unit higher on acceptance, on average scoring 0.116 standard deviations lower on their initial behavioral disengagement. Being rejected in primary school was unrelated to students' initial levels; neither did it predict students' development in behavioral disengagement (*B*_*i*_ = 0.166 [−0.036; 0.368], *p* = .108, *β*_*i*_ = .080; *B*_*s*_ = 0.008 [−0.025; 0.042], *p* = .630, *β*_*s*_ = .025), in contrast to hypothesis 3. More popular students showed higher behavioral disengagement at the end of primary school, yet popularity in primary school was unrelated to students' development of behavioral disengagement over the transition from primary to secondary school (*B*_*i*_ = 0.334 [0.190; 0.477], *p* < .001, *β*_*i*_ = .149; *B*_*s*_ = −0.001 [−0.027; 0.024], *p* = .914, *β*_*s*_ = −.004). The effect on the intercept was relatively small; students who scored one unit higher on popularity, on average scored 0.149 standard deviations higher on their initial behavioral disengagement. This finding was in contrast to hypothesis 4. Girls showed lower behavioral disengagement at the end of primary school compared with boys.

#### The role of ability grouping in disengagement trajectories

Students in the general track differed from students in the pre‐vocational track neither in their initial levels nor development in behavioral disengagement (*B*_*i*_ = −0.059 [−0.150; 0.033], *p* = .211, *β*_*i*_ = −.072; *B*_*s*_ = −0.001 [−0.012; 0.013], *p* = .919, *β*_*s*_ = .006). Students in the pre‐university track had lower initial levels of behavioral disengagement than students in the pre‐vocational track and did not differ from students in pre‐vocational tracks in their development of behavioral disengagement over the transition (*B*_*i*_ = −0.097 [−0.166; −0.027], *p* = .006, *β*_*i*_ = −.148; *B*_*s*_ = −0.001 [−0.012; 0.009], *p* = .816, *β*_*s*_ = −.014). The effect of the intercept was relatively small, with students in the pre‐university track on average scoring 0.148 standard deviations lower on their initial levels of behavioral disengagement than students in the pre‐vocational track. These results are partly in line with hypothesis 5 and 6, as students in the pre‐vocational and pre‐university track differed from each other but not from students in the general track in their initial levels of behavioral disengagement, and we did not find an effect of track on students' development of behavioral disengagement. Repeating the analyses with the general track as the reference category showed that students in the pre‐university track differed from students in the general track neither in their initial levels nor development of behavioral disengagement (*B*_*i*_ = −0.039 [−0.114; 0.035], *p* = .298; *B*_*s*_ = −0.002 [−0.010; 0.006], *p* = .642). The results were similar when considering students' track level as a continuous variable in model 2 (0 = *pre‐vocational*; 1.0 = *general*, and 2.0 = *pre‐university*; *n* = 1564), and when we coded track as one dummy variable, comparing the pre‐vocational track to both other tracks (0 = *general or pre‐university*, 1 = *pre‐vocational*) (calculations of these effects are available upon request).

#### Popular students in pre‐vocational tracks

**FIGURE 2:**
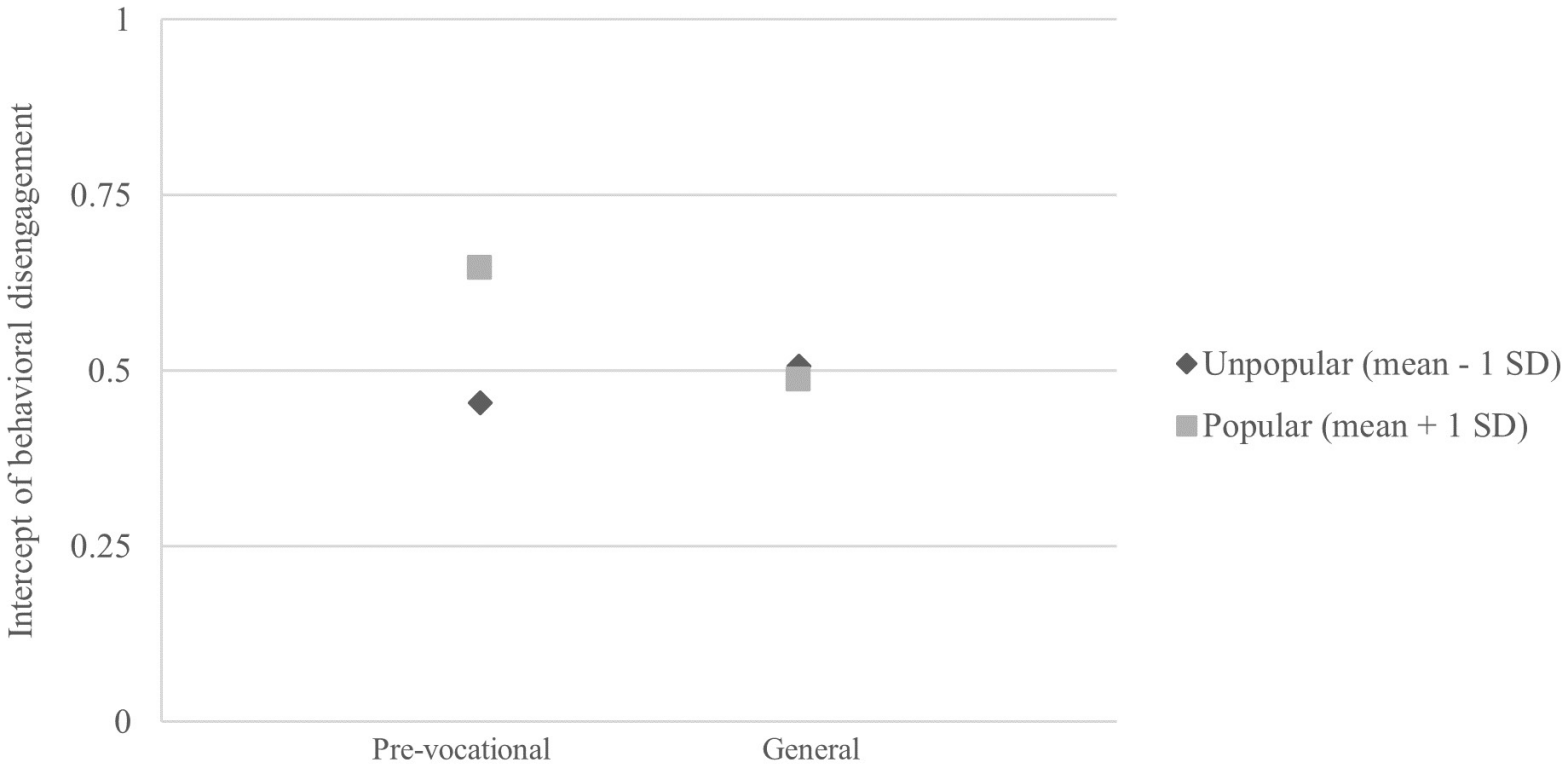
Interaction between popularity and track on the intercept of behavioral disengagement. *Note*: The *y*‐axis is shown from 0 to 1 for visual clarity, the behavioral disengagement scale ranges from 0 to 3.

In order to examine whether popular students in pre‐vocational track would have steeper increases in behavioral disengagement compared to popular students in the other tracks, we added the interaction terms to model 3. Model 3 explained significant variation in the intercept of behavioral disengagement (8.2%, *p* < .001), but not in the slope (1.6%, *p* = .234). Model 3 shows that popular students in the general track had lower initial levels of behavioral disengagement than popular students in the pre‐vocational track, whereas they did not differ in their development in behavioral disengagement (*B*_*i*_ = −0.757 [−1.322; −0.191], *p* = .009, *β*_*i*_ = −.144; *B*_*s*_ = 0.028 [−0.068; 0.124], *p* = .569, *β*_*s*_ = .039). Figure 2 displays the interaction between popularity and track for the intercepts of the pre‐vocational and general tracks. This shows that popular students who ended up in pre‐vocational track had higher behavioral disengagement in primary school than unpopular students who ended up in pre‐vocational track. For students who end up in the general track, there is neglectable difference in behavioral disengagement in primary school between popular and unpopular students. We did not detect differences between popular students in the pre‐university track compared with popular students in the pre‐vocational track in their development in behavioral disengagement (*B*_*i*_ = −0.379 [−0.975; 0.216], *p* = .212, *β*_*i*_ = −.120; *B*_*s*_ = −0.040 [−0.123; 0.042], *p* = .337, *β*_*s*_ = −.094), which is in contrast to hypothesis 7.

## DISCUSSION

In this study, we examined how students' peer status in primary school and ability track in secondary school related to trajectories of behavioral disengagement over the transition from primary to secondary school. Students' behavioral disengagement increased over time, with students reporting the highest levels of disengagement at the end of their last year in primary school. Students' peer status and track level related to behavioral disengagement in primary school, with behavioral disengagement being lower for accepted students and higher for popular students and students ending up in the pre‐vocational track. Peer status (acceptance, rejection, popularity) and track did not relate to students' development in behavioral disengagement over the transition.

### The development of behavioral disengagement over school transitions

On average, students' behavioral disengagement increased over their last year in primary school and the transition to secondary school, in line with our expectations. This increase was small, and the means and additional analyses revealed that the slope was somewhat quadratic. Thus, whereas we theorized that students' behavioral disengagement would mainly increase over the transition to secondary school, students disengaged most at the end of primary school and slightly recovered from this increase in secondary school. The peak in disengagement at the end of the school year may be explained by practical reasons: exams are over and summer holidays are in sight, shifting the focus away from academic goals. This “end‐of‐year‐peak” may particularly occur in the last year of primary school, when students are about to transition to a highly stratified school systems. In the Netherlands, students receive their final track advice for secondary school end of March, which means that academic effort in April, May, and June will no longer contribute to a higher track advice, which may increase disengagement. Moreover, this “end‐of‐year‐peak” may particularly arise in adolescence, when students' social goals increasingly compete with their academic goals (Engels et al., 2017). Future studies should further investigate increases in disengagement at the end of the primary school year to determine the most fruitful way to spend this time and diminish the potential burden of disengaged students for teachers. The slight decrease in disengagement in secondary school does not support the stage‐environment fit theory, which would expect the largest increase in disengagement following the school transition (Eccles et al., 1997). Possibly, the secondary school environment does fit adolescents' needs in some ways. For instance, the new peer environment in secondary school may provide an opportunity for adolescents to improve their peer relationships (Jindal‐Snape et al., 2020; Kinney, 1993).

### The role of prior peer status in disengagement trajectories

Students' peer status related to their behavioral disengagement in primary school, but did not impact their development of behavioral disengagement over the transition to secondary school. Specifically, students who were more accepted by peers in primary school showed lower behavioral disengagement in primary school, in line with our expectations. Despite the effect being small, this supports the notion that being accepted by peers may buffer against disengagement (Ryan et al., 2019). Possibly, accepted students like school better, receive more academic support, and are more comfortable in the classroom, lowering their behavioral disengagement. Being rejected in primary school, however, did not relate to behavioral disengagement in primary school, contrary to our expectations. There may be two opposing effects at play. On the one hand, rejection may lower students' self‐esteem and interest in school, increasing their behavioral disengagement (Furrer & Skinner, 2003). On the other hand, rejected students may focus on academic goals rather than social goals, lowering their behavioral disengagement (Engels, Colpin, et al., 2019; Engels, Pakarinen, et al., 2019). Future studies on peer status and (dis)engagement may contribute to the literature by disentangling these mechanisms. Students who were more popular in primary school showed slightly higher behavioral disengagement in primary school. Thus, contrary to our expectations, popularity may already pose a risk for disengagement in primary school. Popular students may focus on gaining popularity (i.e., social goals) over engaging in school (i.e., academic goals). Showing disengaged behavior may be an effective way to gain popularity, and may be valued by peers as “cool” already in pre‐adolescence (LaFontana & Cillessen, 2010).

Students' peer status in primary school did not relate to students' development in behavioral disengagement over the transition from primary to secondary school, contrary to our expectations. This means that behavioral disengagement increased to the same extent for all students, irrespective of their previous peer status. This parallel development indicates a continuation of differences. Students who were accepted were less disengaged, and students who were popular were more disengaged in primary school, this difference remained in secondary school. At the same time, students' peer status may change in their new peer group in secondary school (Jindal‐Snape et al., 2020). Roughly a quarter of students' primary school peers co‐transition to the same secondary school in the Netherlands (Zwier, Geven, et al., 2023). In turn, students' (possibly changed) peer status in secondary school may impact their disengagement in secondary school (Engels et al., 2017; Engels, Colpin, et al., 2019; Engels, Pakarinen, et al., 2019), rather than their previous primary school peer status. This may partially clarify the limited explained variance in behavioral disengagement, accounting for 5.4% of the variance in the intercept and 0.8% in the slope. Future studies could examine how students' peer status (dis)continues over the transition from primary to secondary school. However, we acknowledge that it is methodologically complex to collect peer nominations also in secondary school, as students transition to many different secondary school classes.

### The role of ability grouping in disengagement trajectories

Students' ability track related to their behavioral disengagement in primary school, but did not impact their development of behavioral disengagement over the transition to secondary school. Specifically, students who ended up in the pre‐university track in secondary school had slightly lower levels of disengagement in primary school than students who ended up in the pre‐vocational track. Students in the pre‐university track did not differ in disengagement in primary school from students who ended up in the general track. This was partly in line with our expectations. Because students are only tracked in secondary school (T3), predicting students' initial behavioral disengagement in primary school (T1) only serves as an approximation for track advice in primary school. It may be that students who were more disengaged in primary school, also achieved lower, resulting in being placed in lower tracks. Another explanation would be that, irrespective of students' achievement, teachers give higher track recommendations to less disengaged students (Timmermans et al., 2016).

Students' ability track in secondary school did not relate to students' development in disengagement over the transition from primary to secondary school. Thus, irrespective of the track students were placed in, students' behavioral disengagement slightly increased towards the end of primary school, and slightly recovered in secondary school. Possibly, the tracked secondary school environment fits adolescents' needs in some ways, relating to the slight recovery in disengagement in secondary school. For instance, groups that are more homogeneous in achievement may permit a focused curriculum, better fitting adolescents' cognitive needs. However, the differences between prospective pre‐vocational students and prospective pre‐university students in primary school, persist in secondary school. Thus, although tracking students on their ability may not further exacerbate these differences, tracking does not neutralize these differences in disengagement either. Thus, placing students who are behaviorally disengaged in primary school in lower tracks does not decrease their disengagement. This is partly in line with the differentiation‐polarization theory, arguing that students in lower tracks develop anti‐school attitudes that increase their disengagement, or, conserve their higher levels of behavioral disengagement (Hargreaves, 1967). In addition, these results imply that higher academic self‐concept and pride of students in lower tracks due to the BFLPE (Holm et al., 2020; Marsh et al., 2008) do not translate into lower behavioral disengagement. Future studies would enrich the literature by examining which aspects of tracking lower students' disengagement (e.g., fitting cognitive needs) and which aspects increase disengagement (e.g., differential school attitudes).

### Popular students in pre‐vocational tracks

Students who were popular in primary school and ended up in the general track showed slightly lower behavioral disengagement in primary school than students who ended up in the pre‐vocational track and did not differ in their development of behavioral disengagement. Popular students in the pre‐university track did not differ from popular students in the pre‐vocational track in disengagement. This was not in line with our expectations. This means that the anticipated increase in behavioral disengagement for popular students was not more pronounced for students in the pre‐vocational track. Yet, students who are popular in primary school and are more behaviorally disengaged, more often end up in the pre‐vocational track, whereas popular students who are less disengaged more often end up in the general track. This may have to do with teachers giving higher track advice to students who they perceive as more confident and more engaged (Timmermans et al., 2016).

### Strengths and limitations

This study has multiple strengths, including following a large sample of adolescents who recently transitioned from primary to secondary school. Moreover, we examined peer status and ability track as important possible predictors of behavioral disengagement, giving an insight into how students differ in their development in behavioral disengagement. Despite the insights gained, this study has multiple limitations.

First, the time points of the measurements are not optimal for measuring a trajectory of behavioral disengagement. Specifically, the second survey was conducted in May–June, roughly 1 month before the end of primary school. This time point diverges from the other time points such that students' behavioral disengagement is higher at the end of the school year. Moreover, we collected data on three time points with only one survey in secondary school, not revealing how behavioral disengagement develops over the first year of secondary education, and limiting the possibility to fit a quadratic model. Although we corrected the unequal intervals by fixing the loadings of the slope factors, and established measurement invariance, future studies should collect data with equal intervals between measurements to meet the assumption of equal intervals.

Second, although this study has the advantage of following students into secondary education, the attrition rate of 45.1% for our secondary school measure (T3) was high. The PRIMS data collection aimed to minimize attrition by collecting multiple contact details from students and offering a lottery as an incentive to participate (Zwier, Lorijn, et al., 2023). Despite the T3 sample being selective on behavioral disengagement, we obtained unbiased parameters by using FIML to handle missing data at random. Future studies following students over school transitions would add to the literature by further minimizing attrition.

Third, we could not evaluate how students' peer status changed in their new peer group in secondary school because we only measured peer status in primary school. It was not feasible to collect peer nominations in secondary school because students transitioned to hundreds of different secondary school classes. Although acceptance and popularity remain relatively stable over school transitions (Brass & Ryan, 2021), peer status may change for some students. For instance, some students may lose friends or popularity, whereas others may gain friendships and enhance their peer status (Jindal‐Snape et al., 2020). Insight into changes in peer status would clarify the co‐evolution of peer status and behavioral disengagement after the transition to secondary school. In addition, including students' peer status in secondary school is promising for explaining more variance in the development of behavioral disengagement.

Fourth, taking a variable‐centered approach limits the possibility to address intra‐individual change, not revealing how many students increase, decrease, or remain stable in their level of behavioral disengagement (Moeller, 2021). We used a variable‐centered approach because this is desired when studying average trajectories of change and predictors for deviations from this trajectory (Laursen & Hoff, 2006). The confidence interval around the slope for our unconditional model revealed that there are individual differences in trajectories, and that some students remained fairly stable but that most increased in behavioral disengagement. To get more insight into these individual differences and increase ergodicity, future studies would complement the findings from our variable‐centered approach by using a person‐centered approach. Future studies could examine within‐person trajectories over time to gain more insight into individual differences (Moeller et al., 2022). To examine group differences in developmental trajectories, we recommend creating latent profiles or classes as a suitable within‐person approach. For instance, peer status groups could be created and compared in their trajectories of behavioral disengagement, with group percentages revealing how many students would increase, decrease, or remain stable (Lorijn et al., 2023).

### Practical implications

The findings of this study have implications for school practice and intervention to reduce students' behavioral disengagement. Our findings show that students' behavioral disengagement increases, with students being most behaviorally disengaged in their last months of primary school. This peak in disengagement is likely form a burden for teachers and increase their workload. Schools and interventions may aim to reduce students' disengagement, or support teachers in other ways to reduce their workload particularly in the last months of the schoolyear.

Reflecting on how students' peer status affects their behavioral disengagement, the findings show that peer acceptance may limit students' behavioral disengagement whereas popularity may be a risk for behavioral disengagement. Therefore, schools and interventions aiming to reduce behavioral disengagement should incorporate students' peer status. They should promote a favorable peer climate characterized by accepting peer relationships, and they should provide alternate ways for students to gain popularity rather than by disengaging. Secondary schools could gain insight into students' peer status in primary school to recognize students who were more popular and less accepted as at risk for continued behavioral disengagement. Schools should use this information to promote students' engagement without stigmatizing students on their previous peer status because this could result in lower teacher expectations and self‐fulfilling prophecies (Rosenthal & Jacobson, 1968).

We found no evidence that tracking reduces students' behavioral disengagement. Students who were more disengaged in primary school more often ended up in lower tracks, possibly because they achieved lower or teachers valued students' disengagement as a reason to be given a lower track recommendation. Primary school teachers may assume that students disengage because they cannot keep up, and this discrepancy between students' cognitive skills and the level of education offered would be bridged by placing them in lower tracks, reducing their disengagement. Yet, behavioral disengagement increased to the same extent for all students, irrespective of their secondary school track. Thus, although placing students in lower tracks may not increase their disengagement, it does not decrease their disengagement either. Notwithstanding the debate on whether students' attributes besides their achievement should determine students' secondary school track (Bennett et al., 1993), our findings would argue that students' disengagement in primary school should not matter for students' track advice, if the goal is to reduce their disengagement.

## FUNDING INFORMATION

This data collection was made possible by a grant awarded to Veenstra et al. (2018) for the project ‘Peer Relations in the Transition from Primary to Secondary school: Social, Behavioral and Academic Aspects of Social Integration’ by the Netherlands Initiative for Education Research (NRO), grant number 40.5.18325.001.

## CONFLICT OF INTEREST STATEMENT

The authors declare no competing interests.

## INFORMED CONSENT

All participants who were involved in PRIMS provided active written parental informed consent.

## Data Availability

PRIMS data will be made publicly available for researchers under certain conditions in 2024, see: Zwier, Lorijn, et al., 2023, DataverseNL, V1.

## APPENDIX 1

### Assumptions for latent growth curve models

Latent growth curve models assume sufficient sample size, repeated measures with equal intervals, a similar type of trajectory for all individuals (i.e., linear or quadratic), and normally distributed outcome variables (Curran et al., 2010). The sample size was adequate to perform LGCA. Specifically, the adequate sample size depends on the research design (e.g., the complexity) and the number of repeated observations per individual, with a preferred sample size of at least 100 (Curran et al., 2010). Thus, 689 repeated observations per individual and a full sample size (i.e., as imputed with FIML) of 1564 is sufficient for our model in terms of estimation and power. The repeated measures of behavioral disengagement have unequal intervals. Loadings of the slope factors are fixed to reflect the unequal intervals to meet this assumption. Moreover, we found measurement invariance, which shows that this assumption is met.

The assumption of individuals following a similar type of trajectory is assessed by the model fit of the unconditional model. Our model assumes a linear trajectory because with three time points only a linear growth model can be estimated (i.e., it is not possible to estimate a quadratic growth model with three time points). The unconditional model showed questionable fit, with marginal fit according to the RMSEA: 0.102 [0.063; 0.146], but acceptable or good fit based on the other fit indices *χ* ^2^ (1, *N* = 1558) = 17.08, *p* < .001, RMSEA = CFI = 0.965, TLI = 0.896, SRMR = 0.026. This may indicate that the slope is (somewhat) quadratic. Moreover, based on the means of behavioral disengagement, increasing from 0.45 to 0.49 in primary school, and decreasing to 0.46 in secondary school, it may be questioned if the slope is quadratic. At the same time, the baseline model estimated a linear slope and indicated that students' average behavioral disengagement increased over time (*B*_*S*_ = 0.005 [0.000; 0.009], *p* = .034). To evaluate if there is a quadratic slope without being able to estimate a quadratic growth model, we estimated a quadratic term while restricting the variance of the quadratic term to zero. This model estimates the linear aspect of the development, controlling for the quadratic aspect. Results showed a significant quadratic effect (*B*_*Q*_ = −0.003 [−0.005; −0.002], *p* < .001). At the same time, the positive slope of the baseline model remained significant (*B*_*S*_ = 0.028 [0.017; 0.039], *p* < .001). All results of model 1, model 2, and model 3 were similar when including the quadratic term. Specifically, only the effect of acceptance in model 3 lost significance, with the *p*‐value increasing from *p* = .054 in the main model to *p* = .058 in the model including the quadratic term. This difference is negligible as we do not evaluate our hypothesis on acceptance in model 3. For all other variables in all models, there were no differences in significance. Thus, while there is some quadratic effect, including a quadratic term does not change the results. Therefore, and based on the majority of the fit indices, we assume that the development is sufficiently linear to estimate a linear slope. Taken together, we decide that the assumption has been met.

Considering skewness, behavioral disengagement is generally non‐normally distributed. In our sample behavioral disengagement was highly positively skewed with most students indicating low behavioral disengagement. Scores on behavioral disengagement showed a leptokurtic distribution (T1: (*n* = 1492) skewness = 1.26 (0.06), kurtosis = 3.77 (0.13); T2 (*n* = 1489) skewness = 1.15 (0.06), kurtosis = 2.57 (0.13); T3: (*n* = 698) skewness = 1.95 (0.09), kurtosis = 8.20 (0.19)). Recoding the scale into three categories through collapsing the third and fourth categories (answers “often” and “always”), yielded very strong correlations between the original and the recoded scale (all *r* = .99). This indicates that recoding the scale into a less skewed scale would not change the results. Therefore, and because behavioral disengagement is generally skewed, the original scale was used. The robust maximum likelihood estimator (MLR) was applied to handle these non‐normal distributions (Curran et al., 2010).
